# Bioinformatics Analysis of Tumor-Associated Macrophages in Hepatocellular Carcinoma and Establishment of a Survival Model Based on Transformer

**DOI:** 10.3390/ijms26199825

**Published:** 2025-10-09

**Authors:** Zhuo Zeng, Shenghua Rao, Jiemeng Zhang

**Affiliations:** School of Mathematics and Physics, Wuhan Institute of Technology, Wuhan 430205, China; 15527853616@163.com (Z.Z.); raoshenghua@stu.wit.edu.cn (S.R.)

**Keywords:** TAMs, survival analysis, concordance index, transformer, scRNA-seq

## Abstract

Hepatocellular carcinoma (HCC) ranks among the most prevalent malignancies globally. Although treatment strategies have improved, the prognosis for patients with advanced HCC remains unfavorable. Tumor-associated macrophages (TAMs) play a dual role, exhibiting both anti-tumor and pro-tumor functions. In this study, we analyzed single-cell RNA sequencing data from 10 HCC tumor cores and 8 adjacent non-tumor liver tissues available in the dataset GSE149614. Using dimensionality reduction and clustering approaches, we identified six major cell types and nine distinct TAM subtypes. We employed Monocle2 for cell trajectory analysis, hdWGCNA for co-expression network analysis, and CellChat to investigate functional communication between TAMs and other components of the tumor microenvironment. Furthermore, we estimated TAM abundance in TCGA-LIHC samples using CIBERSORT and observed that the relative proportions of specific TAM subtypes were significantly correlated with patient survival. To identify TAM-related genes influencing patient outcomes, we developed a high-dimensional, gene-based transformer survival model. This model achieved superior concordance index (C-index) values across multiple datasets, including TCGA-LIHC, OEP000321, and GSE14520, outperforming other methods. Our results emphasize the heterogeneity of tumor-associated macrophages in hepatocellular carcinoma and highlight the practicality of our deep learning framework in survival analysis.

## 1. Introduction

Liver cancer is the sixth most common cancer worldwide and the third leading cause of cancer-related deaths [1]. According to the Global Burden of Disease (GBD) Study 2021, the incidence of liver cancer has risen by 53.7% over the past two decades [2]. Although prognosis has improved over time, the five-year survival rate remains below 20% [3]. Major risk factors include viral infections (such as hepatitis B virus and hepatitis C virus, HBV/HCV), chronic liver diseases (including fatty liver disease and cirrhosis), alcohol misuse, and metabolic disorders (including diabetes).

Macrophages play a central role in balancing immune responses and tissue repair to maintain homeostasis. Once this plasticity is exploited by malignant proliferation, it coordinates multiple interactions in the tumor microenvironment (TME) to drive the evolution of the cancer ecosystem. Although cancer cells exploit the pro-inflammatory function of tumor-associated macrophages (TAMs) to promote tumorigenesis by producing various factors, TAMs involved in immune system recruitment also possess anti-tumor properties [4].

Each TAM subgroup has unique transcriptional features and marker profiles based on the type, stage, and immune composition of the infiltrating tumor [5]. The emergence of single-cell sequencing technology has expanded our understanding of the cellular composition and gene expression characteristics in the tumor microenvironment, enabling us to study intercellular mechanisms and gene expression differences at the single-cell level [6]. By achieving high-resolution visualization of individual cells, single-cell RNA sequencing (scRNA-seq) has played a key role in depicting the diverse immune phenotypes and complex intercellular interactions in hepatocellular carcinoma [7,8].

In recent years, deep learning has made remarkable progress in prediction tasks in the field of survival analysis [9]. However, due to the severe overfitting issues caused by the inherent curse of dimensionality problem in high-throughput sequencing data, accurately predicting prognosis using cancer genomic data remains challenging. Additionally, survival analysis presents unique challenges stemming from the difficulty of handling unobserved and censored samples [10].

Cox regression models use survival outcomes and survival time as dependent variables, enabling simultaneous analysis of multiple factors affecting survival duration and handling censored survival data [11]. The Faraggi–Simon network is regarded as a nonlinear extension of the Cox proportional hazards model [12]. DeepSurv expands upon Faraggi and Simon’s work by employing deep feedforward neural networks to estimate the log-risk function in Cox models, allowing these models to capture increasingly complex relationships between covariates and risk [13]. The FT-Transformer model converts features into embeddings, which are then processed through layers of a Transformer architecture [14]. This approach applies the attention mechanism, which was originally designed for tasks like natural language processing (NLP) to tabular data. The attention mechanism of the model enables it to capture the complex relationships between heterogeneous features.

In this study, we employed single-cell bioinformatics analysis techniques to reveal differences among macrophage subpopulations and explore their association with cancer. Inspired by FT-Transformer and DeepSurv, we performed feature embedding and minimized the negative log-likelihood through a Transformer architecture to predict prognosis.

## 2. Results

### 2.1. Cell Type Identification

**Figure 1:**
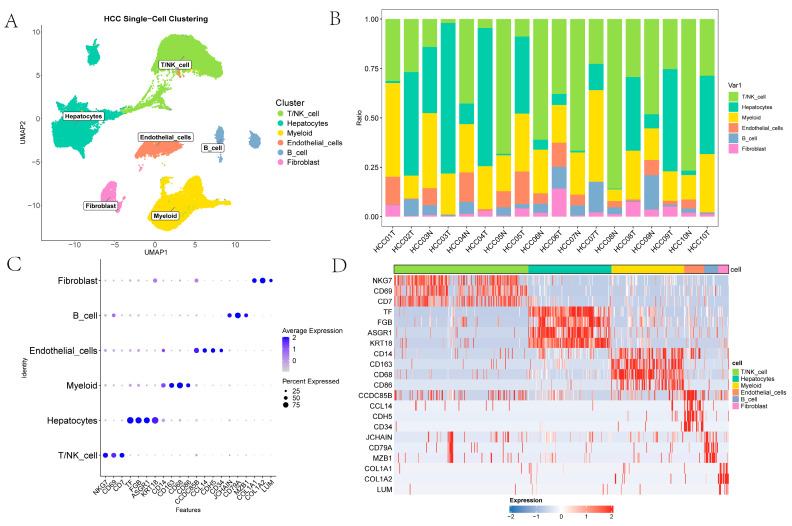
Clustering of HCC scRNA-Seq Data: (**A**) UMAP representation of annotated cell types; (**B**) The proportion of various cell types in different samples; (**C**) A scatter plot showing the expression of genes marking different cell types; (**D**) A heatmap showing the expression of genes marking different cell types.

The single-cell dataset GSE149614 contains four types of tissue samples from 10 patients with primary and metastatic HCC: non-tumor liver (NTL), primary tumor (PT), portal vein tumor thrombus (PVTT) and metastatic lymph node (MLN) tissues [6]. Initially, cells from PVTT and MLN tissues were removed, and the remaining data were processed with the Seurat workflow, yielding 61,356 cells for downstream scRNA-seq analysis. Subsequently, Harmony integration was applied to correct batch effects across samples in GSE149614 datasets [15]. For exploratory visualization, the integrated data were projected into a two-dimensional space using UMAP [16]. Thereafter, FindClusters partitioned the cells into 27 clusters at a resolution of 1. Six major cell types were identified using canonical surface markers: T/NK cells (*NKG7*, *CD69*, *CD7*); myeloid cells (*CD14*, *CD163*, *CD68*, *CD86*); endothelial cells (*CDH5*, *CD34*, *CCDC85B*, *CCL14*); hepatocytes (*TF*, *FGB*, *ASGR1*, *KRT18*); fibroblasts (*COL1A1*, *COL1A2*, *LUM*); and B cells (*JCHAIN*, *CD79A*, *MZB1*) (Figure 1A). As illustrated in Figure 1B, the figure presents the proportions of T/NK cells, hepatocytes, myeloid cells, endothelial cells, B cells and fibroblasts across various samples; this highlights the heterogeneity of the TME. The scatter plots and heatmaps in Figure 1C,D illustrates the expression patterns of the marked genes across different cell types.

### 2.2. Cell-to-Cell Communication Between Six Cell Types

**Figure 2:**
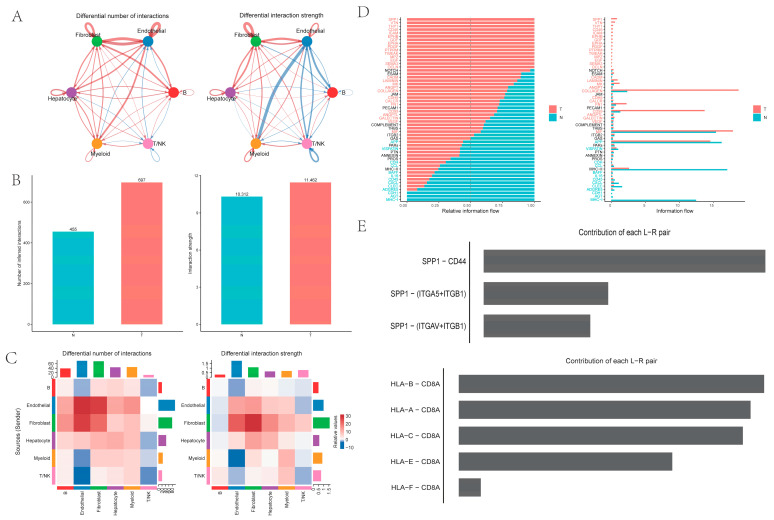
Intercellular communication among six cell types: (**A**) Comparison of intercellular communication quantity (left) and intensity (right) between tumor and non-tumor tissues; blue indicates reduced intensity in non-tumor tissue relative to tumor tissue; (**B**) Bar charts showing overall quantity (left) and intensity (right); (**C**) Heatmap comparing the quantity (left) and intensity (right) of intercellular communication between specific ligands and receptors; (**D**) Differences in pathway intensity between tumor and non-tumor tissues (*y*-axis in black indicates no difference between the two groups); (**E**) The two pathways with the greatest differences between tumor and non-tumor tissues, involving specific ligand-receptor pairs.

Intercellular analysis revealed that both the number and the strength of cell interactions were higher in PT tissues from HCC than in NTL tissues from HCC. Notably, ligand–receptor pairs between myeloid cells and endothelial cells exhibited significantly higher interaction intensity in NTL tissues compared with PT tissues, representing the most pronounced change in interaction strength (Figure 2A–C). Pathway-level analysis showed that the *SPP1* pathway was most significant in PT tissues, with the strongest SPP1–CD44 ligand–receptor interaction, whereas the *HLA-I* pathway was most significant in NTL samples (Figure 2D,E).

### 2.3. Myeloid Cells Single-Cell Atlas

**Figure 3:**
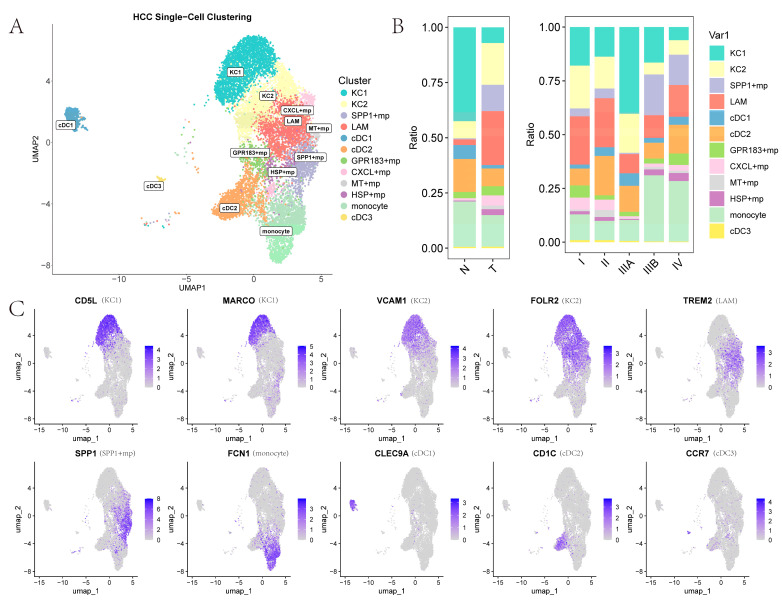
Myeloid cell subpopulations: (**A**) UMAP representation of samples, clusters, and annotated cell types after reclustering myeloid cells and performing harmony. Each point represents a cell; (**B**) Proportions of various cell types in normal liver tissue, primary tumor tissue, different cancer stages, and different samples; (**C**) Gene expression profiles of marker genes for each types of myeloid cell subpopulation.

To explore the subtle differences in TAMs, we performed secondary clustering and defined myeloid subpopulations into four categories: Kupffer cells (KCs), other macrophages, monocytes, and dendritic cells (DCs) (Figure 3A). Clusters 0, 1, and 11 were designated as KCs1 due to the high expression of canonical KC markers *CD5L*, *CETP*, and *MARCO* [17]. Clusters 2 and 5, which exhibit high expression of *TREM2*, *GPNMB*, and *CD9* but low expression of *SPP1*, were designated as lipid-associated macrophages (LAMs) [18]. Cluster 4, showing high *SPP1* expression but low *TREM2* expression, was designated *SPP1*+ macrophages [19]. Clusters 7, 8, and 10 lie between KCs1 and LAMs in the UMAP plot; they display partial expression of *CD5L*, *CETP*, and *MARCO*, along with high expression of *FOLR2* and *C1Q* complex genes (*C1QA*, *C1QB*, *C1QC*), and are therefore designated as KCs2 (*FOLR2* and *C1Q* complex genes appear among KC markers in some literature) [17]. Clusters 6, 9, 15, and 18, which exhibit notably high *FCN1* expression, are designated as monocytes. The designation of clusters 13, 3, and 24 as cDC1, cDC2, and cDC3 is based on high expression of *CLEC9A*, *CD1C*, and *CCR7*, respectively [20]. The remaining clusters, 16, 21, and 23, are designated as *CXCL*+ macrophage, *HSP*+ macrophage, and *MT*+ macrophage, respectively, due to high expression of homologous genes [21]. Finally, cluster 12 shows no clearly identified marker genes in the existing literature and no highly expressed genes with known functions; it is labeled *GPR183*+ macrophage (Supplementary File S1). Additionally, the expression of marker genes for certain myeloid cell subtypes on the UMAP plot is shown in Figure 3C.

By examining the proportions of macrophages across tissues and disease stages (Figure 3B), several patterns emerge. When comparing the KC subtypes, KCs1 is enriched in NTL tissues, whereas KCs2 is relatively more abundant in PT tissues. Moreover, KCs are more prevalent in early-stage cancer than in late-stage disease, consistent with the general observation that resident KC populations are diminished or absent in liver diseases, with some exception in stage 3. The LAMs and *SPP1*+ macrophage groups also show significantly higher proportions in PT than in NTL tissues, and *SPP1*+ macrophages are largely confined to late-stage HCC PT tissues. Additionally, macrophage groups with homologous marker genes–*CXCL*+ macrophages, *MT*+ macrophages, and *HSP*+ macrophages exhibit substantially higher proportions in PT than in NTL tissues.

### 2.4. Identification of Co-Expression Modules in Myeloid Cells

**Figure 4:**
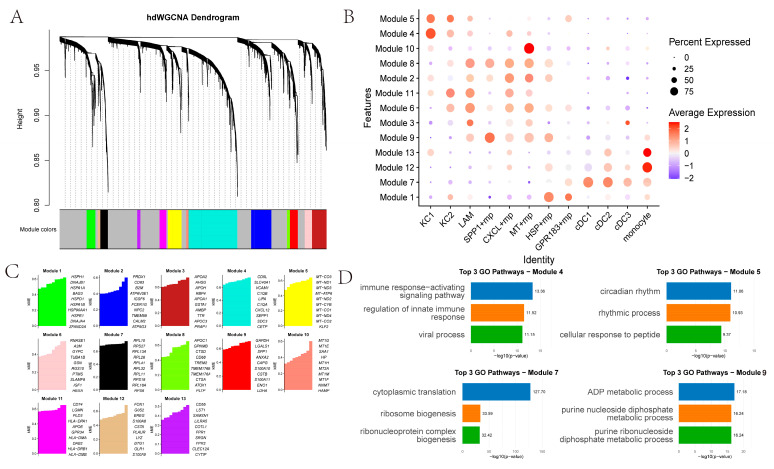
hdWCGNA: (**A**) hdWGCNA dendrogram, with gray modules composed of genes not assigned to any co-expression modules; (**B**) Module expression in different myeloid cells; (**C**) Top 10 genes in each module; (**D**) Top three GO pathways involved in different modules.

We identified 13 co-expression modules across 12 myeloid cell types (Figure 4A–C). KC1 and KC2 showed higher correlation with Modules 4 and 5. Module 4’s top GO terms relate to immune response-activating signaling pathways, regulation of innate immune responses, and viral processes (Figure 4D). Module 5’s top GO terms pertain to circadian rhythm, rhythmic processes, and cellular responses to peptides. SPP1+ macrophages are significantly associated with Module 9, whose top GO enrichments pertain to ADP metabolism. Other macrophage subsets link to Modules 8, 2, 11, 6, 3 and 9. In addition, *MT*+ macrophages are significantly associated with Module 10, in which the top 10 genes are metallothionein (*MT*) family genes. *HSP*+ macrophages are significantly associated with Module 1, and the top 10 genes are heat-shock protein (*HSP*) family genes. Monocyte-like cells associate with Modules 12 and 13, while DCs (cDC1, cDC2, cDC3) associate with Module 7.

### 2.5. Trajectory of Myeloid Cells

**Figure 5:**
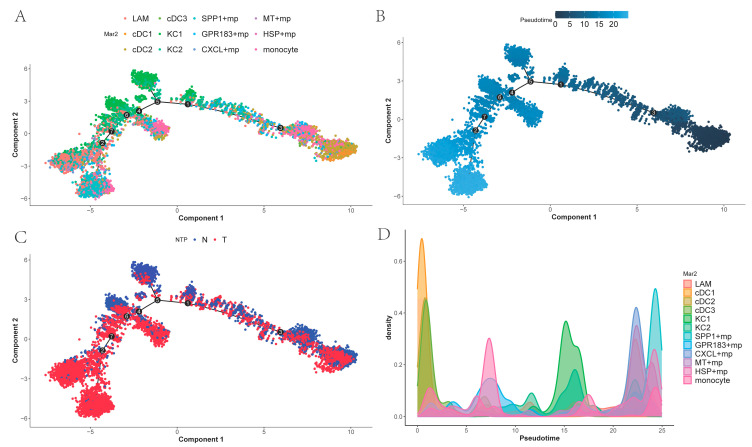
The differentiation trajectory of myeloid cells (the numbers inside the black circles represent different state nodes of the cells): (**A**) *SPP1*+ macrophages is at the terminal stage of differentiation, while DCs is at the starting point of differentiation; (**B**) Temporal representation of the divergence trajectory; (**C**) Different tissue representations of differentiation trajectories; (**D**) Density plots of different myeloid cells over time.

To investigate temporal differences in differentiation among tumor-associated macrophages and the differentiation order of related cell states, we performed cell trajectory analysis of the myeloid subpopulations using Monocle 2 and ordered cells along pseudo-time. As shown in (Figure 5A,B), DCs occupy the early stage of differentiation, and monocytes, lacking subpopulation differentiation, are scattered across various stages of differentiation. KCs represent an early differentiation stage within macrophages excluding dendritic cells and monocytes. While *SPP1*+ macrophages are found at the terminal end of the trajectory, *GPR183*+ macro-phages, *CXCL*+ macrophages, *MT*+ macrophages, and *HSP*+ macrophages also appear in later portions of the trajectory, combined with Figure 5C. This suggests that these macrophages may represent a distinct functional subtype of cells generated during cancer progression. Figure 5D shows the density plots of different myeloid cells over time.

### 2.6. Estimate the Relative Proportions of Macrophage Subpopulations in TCGA-LIHC

**Figure 6:**
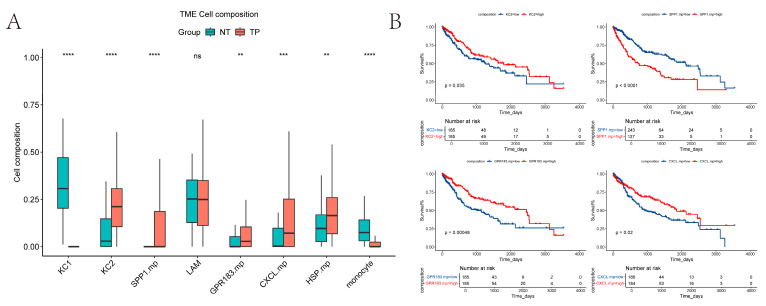
CIBERSORT and KM-plot: (**A**) Inference of the distribution of multiple macrophage subpopulations in tumor and normal samples in the TCGA-LIHC dataset. (Some cells were excluded.) The *p*-values shown are from the Wilcoxon test. ns, no significant difference; ** *p* < 0.01; *** *p* < 0.001; **** *p* < 0.0001; (**B**) Relationship between overall survival and cell proportion in the TCGA cohort (*p* < 0.05), with time calculated in days. The *p*-values shown are from the log-rank test.

We used CIBERSORT to estimate the relative proportions of macrophage subpopulations in TCGA tumor versus normal samples (Figure 6A). Because *MT*+ macrophages were present at very low numbers (cells < 200), they were excluded from the analysis. Our results show that KCs1 and monocyte-like cells are more abundant in normal tissue, whereas KCs2 and *SPP1*+ macrophages are more prevalent in tumor tissue. These patterns are broadly consistent with our previous single-cell sequencing results on normal and tumor tissues (LAMs excluded).

To assess the prognostic value, we analyzed overall survival in the TCGA cohort using the “survminer” R package (version 0.4.9) [22]. The proportions of KCs2, *SPP1*+ macrophages, *GPR183*+ macrophages, and CXCL+ macrophages all showed significant associations with OS (log-rank *p* < 0.05). However, the direction of the associations differed: high *SPP1*+ macrophages infiltration correlated with poorer prognosis, while lower infiltration of KCs2, *GPR183*+ macrophages, and *CXCL*+ macrophages was associated with worse outcomes (Figure 6B).

### 2.7. Survival Analysis with Other Model

We performed subsequent survival analyses using bulk RNA-seq datasets TCGA-LIHC, OEP000321, and GSE14520, benchmarking them against other prognostic models. Because the relative abundances of KCs2, *SPP1*+ macrophages, *GPR183*+ macrophages, and *CXCL*+ macrophages were significantly associated with overall survival differences, we used their marker genes (539 in total See Supplementary File S2) for survival analysis. Presented in Table 1 are the sample size, final number of feature genes, and deletion ratio for each dataset.

For each dataset, we trained and evaluated our model (ZZFormer) and other baseline models through 20 independent rounds of five-fold cross-validation. In each fold of every round, the data was divided into five subsets: one subset was assigned as the test set, another as the validation set, and the remaining three were used for training. For models that did not require a validation set (i.e., RSF and GBM), all four non-test subsets were combined to form the training set. To enhance robustness, the entire process was repeated 20 rounds using different random seeds for data splitting, yielding a total of 5-fold × 20 rounds = 100 independent training-validation-testing runs.

Regarding hyperparameter settings for ZZFormer and other baseline models, we performed grid search on key hyperparameters using one round of five-fold cross-validation. For each candidate hyperparameter set, we computed the average sum of C-index scores across the training and validation sets in all five folds, selecting the configuration with the best performance. This optimal hyperparameter set was then applied to all subsequent five-fold cross-validation runs, repeated with multiple random seeds. For our model, the determined hyperparameters are: embedding length = 64; number of embeddings = floor (number of genes/embedding length); num_heads = 8; Transformer blocks = 2; learning rate = 0.002; dropout = 0.1 [23]; optimizer = Adam with L2 regularization (weight decay) [24,25]. DeepSurv is invoked through the “pycox” python package (version 0.3.0) [26], Deep Survival Machines (DSM) through the “auton-survival” python package (version 0.1.0), and Random survival forests (RSF) and Gradient Boosting Machine (GBM) through the “sksurv” python package (version 0.24.0) [27]. Model performance was evaluated using a predefined metric (C-index); the results are shown in Table 2, with the best-performing model across the three datasets highlighted in gray.

On the TCGA-LIHC dataset, ZZFormer achieved a C-index of 0.654 ± 0.059, significantly higher than DeepSurv (0.633 ± 0.051) and DSM (0.606 ± 0.065), and slightly higher than the non-neural-network baselines RSF (0.649 ± 0.061) and GBM (0.652 ± 0.054). On GSE14520, ZZFormer reached 0.648 ± 0.066, outperforming other neural-network baselines (DeepSurv 0.606 ± 0.071; DSM 0.610 ± 0.075) and RSF/GBM (0.617 ± 0.073 and 0.602 ± 0.060, respectively). On OEP000321, ZZFormer attained 0.689 ± 0.073, continuing to surpass DeepSurv (0.640 ± 0.079) and DSM (0.641 ± 0.082) and also higher than RSF (0.672 ± 0.071) and GBM (0.655 ± 0.064) (Table 2). Taken together across the three datasets, ZZFormer delivers superior high-dimensional survival-prediction performance with strong generalization, outperforming most baselines, including both neural-network and non-neural-network models.

### 2.8. Feature Importance

**Figure 7:**
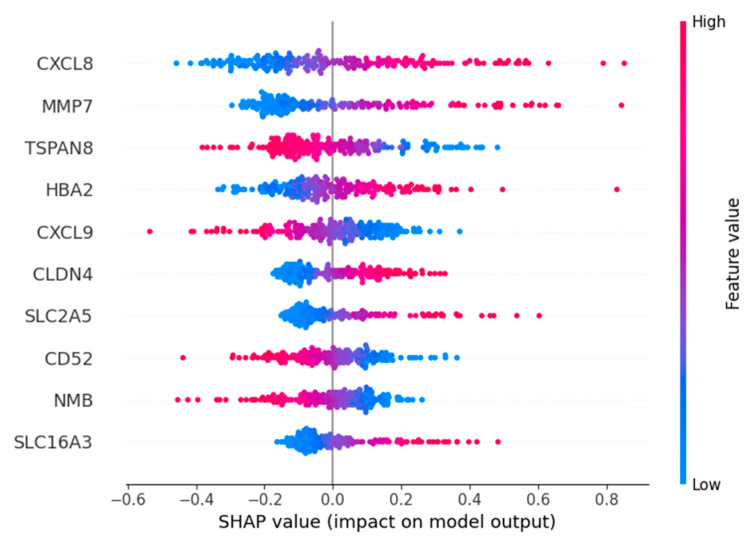
SHAP value for top 10 important genes: Red features make the predicted value larger (similar to positive correlation), blue makes the predicted value smaller, and purple is close to the mean. The wider the color area, the greater the influence of the feature.

Based on the importance ranking of Shapley Additive Explanations (SHAP) [28], the top five genes with the highest importance ranking among the four macrophage marker genes are *CXCL8*, *MMP7*, *TSPAN8*, *HBA2*, and *CXCL9* (Figure 7).

Expression and role for the *CXCL8* family of chemokines in acute and chronic inflammatory conditions and cancer. These molecules may be, however, relevant for host immune responses against certain infections [29]. MMP-7 can regulate the occurrence and development of cancer and mediate the proliferation, differentiation, metastasis and invasion of various types of cancer cells through multiple mechanisms [30]. *TSPAN8* is associated with tumor growth and metastasis. Overexpression of *TSPAN8* promotes the expression of stem cell markers such as *ALDH1A1*, increases the proportion of CD44 +/CD24 − cells, and enhances the expression of pluripotent transcription factors (including *SOX2*, *OCT4* and *NANOG*) [31]. *HBA2* encodes hemoglobin α2 and is an erythroid gene; it is not related to macrophage biology and likely appears here as a redundant/erythroid-specific signal [32]. Chemokine *CXCL9* is a member of the *CXC* family and plays a significant role in the chemotaxis of immune cells. The *HBx* protein can induce the transcription of *CXCL9* by activating NF-κB that binds to its promoter, and *CXCL9* promotes the migration of white blood cells in the liver infected with hepatitis B virus. Moreover, an increasing amount of evidence indicates that *CXCL9* acts as a cancer-promoting factor in various types of cancer [33]. The top five important genes, except for *HBA2*, are all related to cancer and have the potential to be targeted genes, which indicates the feasibility of our model in screening important characteristic genes.

## 3. Discussion

At the single-cell level, we performed stringent quality control, batch correction, and dimensionality reduction on scRNA-seq data derived from HCC PT and adjacent NTL, followed by clustering analysis. This approach identified six major cellular clusters and nine macrophage-associated subclusters, delineating the detailed composition of myeloid cells within the HCC microenvironment. Secondary clustering analysis revealed that hepatic sinusoidal macrophages (KCs) exist in distinct transcriptional states: KC1 was more abundant in NTL, whereas KC2 was relatively enriched in PT. Notably, both LAMs and *SPP1*+ macrophages demonstrated significant enrichment in tumor tissues. Moreover, the presence of *SPP1*+ macrophages was primarily associated with the advanced stages of tumor progression. In addition, macrophage subpopulations with unique functional signatures—designated as *CXCL*+, *HSP*+, and *MT*+—were identified. Collectively, these findings underscore the substantial heterogeneity and plasticity of TAMs in HCC, indicating that these distinct subpopulations may exert differential roles in tumorigenesis, immune regulation, and metabolic reprogramming.

In addition, cell-to-cell communication analysis with CellChat revealed that PT tissues exhibited a higher overall number and strength of cell–cell interactions compared to NTL tissues. Notably, communication between myeloid cells and endothelial cells appeared relatively attenuated in PT. At the signaling pathway level, the *SPP1* axis emerged as the most prominent in PT, where the SPP1-CD44 ligand-receptor pair demonstrated the strongest interaction, implicating this pathway in tumor-associated inflammation, cellular migration and adhesion, and immunosuppression. Strikingly, *SPP1*+ macrophages and the SPP1-CD44 axis exhibit conserved features across diverse cancers. In contrast, the *HLA-I* pathway displayed heightened activity in NTL, reflecting relatively preserved antigen presentation and immunomodulatory functions. Simulated cell trajectory analysis using Monocle2 delineated a differentiation pathway: DCs were positioned early in the trajectory, KCs1/KCs2 occupied an intermediate stage, and specialized macrophage subsets (*SPP1*+, *GPR183*+, *CXCL*+, *MT*+, *HSP*+) resided predominantly later in the trajectory. Weighted gene co-expression network analysis (hdWGCNA) further connected myeloid subsets to distinct functional modules: KC was primarily associated with modules related to immune activation, innate immune regulation, and circadian rhythms. *SPP1*+ macrophages were significantly enriched in modules involving ADP/nucleoside diphosphate metabolism and exhibited elevated activity in pathways such as glycolysis, HIF-1 signaling, amino acid synthesis, and carbon metabolism. *MT*+ and *HSP*+ macrophages were strongly linked to metallothionein- and heat shock protein-related modules, suggesting a central role for oxidative stress mitigation and protein homeostasis maintenance in tumor adaptation. Finally, CIBERSORT was employed to estimate the relative proportions of these macrophage subsets in TCGA-LIHC samples. These proportions significantly correlated with overall survival (OS), particularly the abundances of KC2, *SPP1*+, *GPR183*+, and *CXCL*+ macrophages. Importantly, high infiltration of *SPP1*+ macrophages was an extremely significant predictor of poor prognosis (*p* < 0.0001).

We use key macrophage marker genes to build and evaluate a Transformer-based survival model across three transcriptomic cohorts: TCGA-LIHC, OEP000321, and GSE14520. The model uses linear embedding, multi-head self-attention, and learnable class tokens, with Cox partial likelihood as the optimization objective to effectively capture gene–gene dependencies and nonlinear risk patterns. Across datasets, the model achieved the highest or tied-highest C-index, approximately 0.65437 in TCGA-LIHC, 0.68922 in OEP000321, and 0.64752 in GSE14520, outperforming representative baselines such as DeepSurv and DSM in a robust manner. This suggests that the attention mechanism can enhance feature selection and feature interaction modeling in high-dimensional gene expression data, improving risk discrimination and cross-cohort generalization. Notably, interpretability analyses indicate that the model captures genes highly associated with patient survival. Our results provide novel directions for subsequent studies.

Nevertheless, several limitations warrant consideration. First, the sample set comprises a limited number of patients, which may not fully capture the heterogeneity of macrophage subpopulations in hepatocellular carcinoma. The tumor microenvironment of hepatocellular carcinoma is composed of a series of complex components, and multiple factors may influence the immune environment. Second, the proposed model is unimodal and based on a single data modality; its stability and generalizability have yet to be fully established and may be improved by incorporating additional data types and validating in independent cohorts. Finally, through SHAP analysis, we identified the hemoglobin gene *HBA2* as making a significant contribution to the model. This gene is a characteristic marker for *GPR183*+ macrophages and may be influenced by red blood cell phagocytosis or technical artifacts in the data. This suggests that our current feature set has limitations in specificity and requires more refined feature selection.

## 4. Materials and Methods

### 4.1. Data Collection and Processing

Single-cell transcriptomic data were obtained from GSE149614 in the Gene Expression Omnibus (GEO) and used to construct a liver macrophage atlas from scRNA-seq, enabling screening for survival related macrophage genes. We also downloaded bulk expression data for survival analysis: TCGA-LIHC and OEP000321 [34], as well as GEO dataset GSE14520 [35,36]. To evaluate the prognostic impact of the target genes, survival models were built using these three datasets.

### 4.2. Single-Cell RNA-Seq Analysis

Single-cell RNA sequencing data were processed and normalized using the Seurat R package (version 5.0.3), following stringent quality-control measures [37]. Cell viability was assessed based on feature counts and mitochondrial gene content. Cells were excluded if they were low quality or dead (genes detected in fewer than 3 cells; cells with <200 or >6000 detected genes; and those with >20% mitochondrial gene content). After QC, we used FindVariableFeatures to identify highly variable genes and applied principal component analysis (PCA) for dimensionality reduction. For clustering, we performed Uniform Manifold Approximation and Projection (UMAP) on the top 20 principal components, and marker genes were identified with FindAllMarkers. Cell-type annotation was supported by the CellMarker 2.0 database and existing literature to ensure accurate categorization [38].

### 4.3. TAM Subset Analysis

Considering the heterogeneity of TAMs, we performed secondary clustering of myeloid cells. This allowed us to examine the distribution and expression profiles of characterized genes in each macrophage subpopulation, thus providing insight into their specificity. Our annotation of subclustering followed a two-step approach. First, subtype-specific marker genes were primarily derived from published TAM-HCC studies. For each cluster, the top 20 differentially expressed genes (DEGs) with the smallest *p*-values were identified using FindAllMarkers. Most subclusters could be annotated by matching these top 20 genes to established macrophage markers reported in the literature. For clusters lacking obvious marker genes, if many top-20 genes belonged to the same gene family, the cluster was named with a gene-family prefix; otherwise, it was named after the top-ranked gene. The top 20 genes for each cluster are detailed in Supplementary File S1.

### 4.4. Inferring Intercellular Communication Networks

CellChat (version 1.6.1) is an R package for inferring intercellular communication networks from single-cell transcriptomic data and is widely used to reveal signaling patterns among different cell types within tissues. By leveraging curated ligand–receptor interaction resources (e.g., CellChatDB) and estimating the communication probability or signal strength between cell populations, CellChat can identify signaling pathways that change significantly under specific biological conditions (such as disease, developmental stage, or treatment) and compare intercellular communication across samples or conditions [39].

### 4.5. Co-Expression Modules in Myeloid Cells

In scRNA-seq data, hdWGCNA can help to identify cell-type specific gene modules and further explore the relationship between these modules and cell state or disease [40]. The core goal of hdWGCNA is to construct weighted co-expression networks of genes between cells and identify gene modules. Finally, modules can be analyzed based on their eigenvalues correlated with cell type or phenotypic data (e.g., disease state, developmental stage, etc.).

### 4.6. Single-Cell Trajectory Analysis

Single-cell trajectory analysis aims to infer developmental trajectories at the single-cell level. By analyzing single-cell RNA-seq data, it reveals the dynamic changes cells undergo during development or differentiation. Cell trajectory analysis tools monocle R package (version 2.32.0) [41], use dimensionality-reduction methods (e.g., t-SNE or UMAP) to project high-dimensional data into two- or three-dimensional space for visualization, generating pseudotime trajectories that depict the distribution of cells along developmental progress. This approach identifies genes that change with pseudotime and validates the findings by integrating known biology and supporting experimental data.

### 4.7. Gene Enrichment Analysis

Gene Enrichment Analysis (GEA) is a widely used bioinformatics method for interpreting the biological significance of functions or features that appear to be significantly enriched in a genome or a set of genes. Gene Ontology (GO) is a standardized language and classification system for describing gene function and genomics data [42]. It is a classification system for annotating the functions, processes, and cellular components of genes and proteins. Kyoto Encyclopedia of Genes and Genomes (KEGG) is a database resource that contains the functions and information of biological systems, providing information on genomes, chemicals, metabolic pathways, diseases, and drugs [43]. GO- and KEGG-based enrichment analyses of gene sets corresponding to different macrophage subtypes can illuminate the biological functions and interactions associated with these genes, providing an initial view of the functional profiles and differences among macrophage subtypes.

### 4.8. CIBERSORT Immune Infiltration

We used CIBERSORT R package (version 0.1.0) for immune infiltration analysis. CIBERSORT, a tool for immune infiltration analysis tool based on linear support vector regression (LSVR). Through the expression profiles of macrophage-subtype-specific DEGs after screening, the bulk gene expression data from TCGA LIHC tumor and adjacent normal tissues were deconvoluted using the R script provided in the guidelines to estimate the abundances of different TAM populations. The criteria for defining macrophage-subtype-specific DEGs were FDR < 0.05, min.pct = 0.25, and |log2FC| > 1.0 [44].

### 4.9. Our Model Diagram

**Figure 8:**
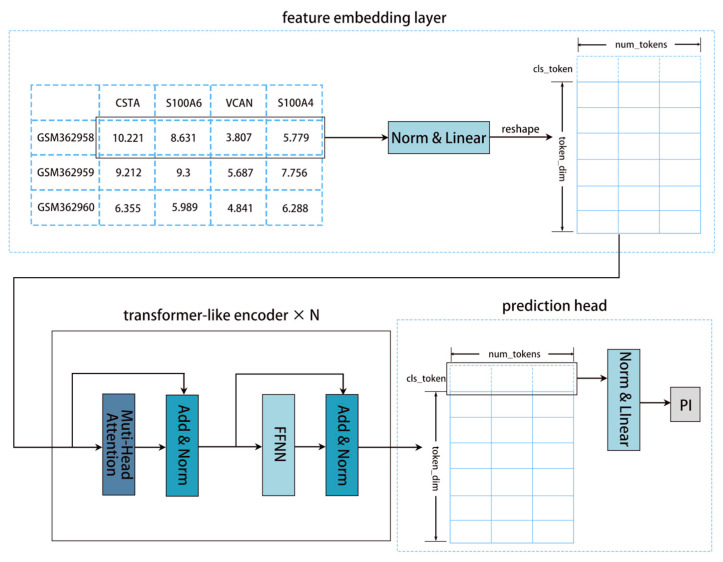
Our model (ZZFormer) diagram: Inputs pass through an embedding layer, a Transformer-like encoder layer, and a prediction head to generate the predicted prognosis index.

Our model (ZZFormer) is a Transformer-based model tailored for survival analysis with high-dimensional feature inputs [45]. The architecture comprises three components: (1) a feature embedding layer, (2) sum transformer-like encoder layers, and (3) a prediction head. The detailed model architecture is shown in Figure 8.

Our feature embedding layer first processes the input features $x\in R^{B\times num\_features}$ through LayerNorm and then linearly projects them into a fixed number of tokens (num_tokens). Each token has token_dim dimensions, producing a sequence $X\in R^{B\times num\_tokens\times token\_dim}$. Following the ViT model, a learnable CLS token is prepended to the sequence to interact with all elements and aggregate global information. This composite sequence is then fed into the Transformer architecture alongside the rest of the model components for prognosis prediction [46].

**Figure 9:**
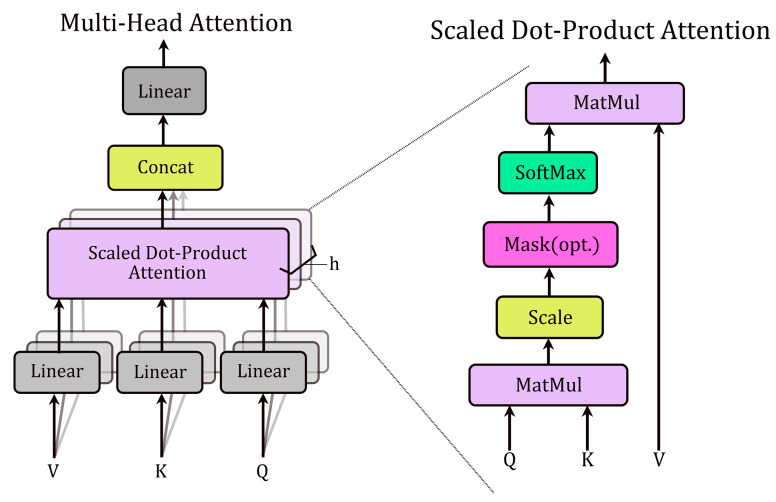
Network architecture diagram of Multi-Head Attention and Scaled Dot-Product Attention.

Each transformer-like encoder layer consists of a multi-head self-attention layer and a feedforward layer. Additionally, layer normalization and residual connections are used to stabilize training. Self-attention enables each position in the sequence to attend to all other positions, effectively capturing dependencies within the sequence. Each attention head employs learnable linear projections to generate three components from the input sequence X: key ($K\;\in \;R^{m\times k}$), query ($Q\;\in \;R^{m\times k}$), and value ($V\;\in \;R^{m\times k}$) matrices (Figure 9). The self-attention operation computes pairwise attention scores via the dot product of *Q* and *K*, scales them by $1/{\left(d_{k}\right)}^{\frac{1}{2}}$ (where $d_{k}$ is the key dimension), applies a softmax normalization, and uses the resulting weights to compute a weighted sum of V:

(1) $${Attention}_{\left(Q,K,V\right)}=softmax\left(\frac{QK^{T}}{{\left(d_{k}\right)}^{\frac{1}{2}}}\right)V$$

This mechanism enables the Transformer to learn dependencies between input features by dynamically weighting their contributions.

After processing through the transformer-like encoder layers, the prediction head uses the CLS token to obtain the prognosis. The CLS token is normalized and passed through a linear layer to produce the Prognosis Index (PI). A higher PI indicates a poorer prognosis, while a lower PI indicates a better prognosis.

We use SHAP to interpret the feature importance of the model. SHAP is a model interpretation framework based on cooperative game theory, which can provide consistent local feature contribution values for prediction models and help understand which input feature contributions the predictions of samples come from.

### 4.10. Loss Function

Cox proportional hazards model is a semiparametric regression model proposed by British statisticians, and it is a frequently employed survival model in survival analysis [11]. The Cox proportional hazards model can be expressed as

(2) $$\begin{array}{c} h\left(t,x\right)=h_{0}\left(t\right)exp\left(\beta^{T}X\right) \end{array}$$

where $h_{0}(t)$ is the baseline risk function served as the part of the nonparametric model. X is the relevant factor that may affect the survival time, called a covariate, and β is the weight coefficient of the variable X. $r(a)=\;\beta^{T}X$ is called the risk function and is a parametric model. Therefore, the Cox model is called as a semiparametric model. But its hazard function r(a) is a linear function, which cannot fit the complex nonlinear scene between variables in reality. To optimize the model and predict patient risk, the gradient descent approach is used to minimize the negative log partial likelihood loss function Cox during the training, which can be defined as follows:

(3) $$\begin{array}{c} L_{cox}=-\sum_{i:\delta_{j}=1}\left(r_{i}-log\sum_{j:t_{j}\geq t_{i}}\exp\left(r_{j}\right)\right) \end{array}$$

### 4.11. Baseline Methods

With five-fold cross-validation, we compared the C-index of our model with that of the following state-of-the-art baseline methods.

- RSF: this ensemble model is similar to random forest and uses survival trees to predict the ensemble cumulative risk function [47].
- GBM: this is an ensemble model based on gradient boosting, which builds a base learner with a greedy strategy [48].
- DeepSurv: this was the first neural network model to outperform the CPH model. It uses neural networks to fit the relationship between covariates and the log risk [13].
- DSM: use neural networks to model potential events through a mixture of fixed (K) parameter distributions [49].

### 4.12. Hyperparameter Optimization

The complete search scope for all models is as follows:

- ZZFormer: token_dim: [32, 64], num_heads: [4, 8], num_blocks: [1, 2], dropout: [0.1, 0.3], Learning_Rate: [0.01, 0.002], L2: [0.05, 0.1, 0.3].
- Random Survival Forest (RSF): n_estimators: [100, 500], max_depth: [3, 5, None], min_samples_split: [0.01, 0.02, 0.05], min_samples_leaf: [0.005, 0.01, 0.02], max_features: [‘sqrt’, ‘log2’, None].
- Gradient Boosting Machine (GBM): learning_rate: [0.05, 0.1], n_estimators: [100, 200], max_depth: [3, 5], min_samples_split: [0.01, 0.05], min_samples_leaf: [0.005, 0.01], subsample: [0.9, 1.0], max_features: [‘sqrt’, ‘log2’].
- DeepSurv: num_nodes: [[64], [64, 64]], dropout: [0.1, 0.3], lr: [0.1, 0.03, 0.01]
- Deep Survival Machines (DSM): k: [3, 4, 6], distribution: [‘LogNormal’, ‘Weibull’], learning_rate: [0.001, 0.0001], layers: [[], [64], [64, 64]].

### 4.13. Model Evaluation Metrics

Using the same evaluation metrics as in article [50], we evaluated each model by the C-index. C-index is a widely used ranking metric for evaluating the discriminative ability of a survival analysis model, it counts concordant pairs between the predicted risk score. The range of C-index is from 0 to 1. The larger its value, the stronger the ability to distinguish the risks of samples.

(4) $$\begin{array}{c} C-index=\frac{\sum_{i,j}1\left\{{ƞ}_{i}<{ƞ}_{j}\right\}1\left\{T_{i}>T_{j}\right\}\delta_{j}}{\sum_{i,j}1\left\{T_{i}>T_{j}\right\}\delta_{j}} \end{array}$$

Here ${ƞ}_{i}$ and $T_{i}$ are the predicted risk score and overall follow-up time for patient i, respectively. The terms $1\left\{...\right\}$ and $\delta_{j}$ are both indicators: $1\left\{...\right\}$ takes value 1 if the argument in $\left\{...\right\}$ is true and 0 otherwise;${\;\delta }_{j}$ takes value 1 if the death of patient j is observed and 0 if patient j is censored.

## 5. Conclusions

This study employs single-cell bioinformatics techniques to characterize macrophage subpopulations and explore their relationship with cancer progression. We integrated 539 marker genes from four prognostically significant tumor-associated macrophage subtypes (KCs2, *SPP1*+, *GPR183*+, and *CXCL*+ macrophages) and established a corresponding deep survival model. The model employs feature embedding techniques, undergoes transformer-like processing, and predicts prognostic indices by minimizing the negative log-likelihood function in the prediction head. Robust performance was demonstrated across three independent RNA sequencing cohorts (TCGA-LIHC, OEP000321, and GSE14520), validating the potential application of the identified macrophage-associated gene signatures in prognostic assessment.

Several limitations should be acknowledged: First, to directly assess the independent predictive value of TAM-specific biological features, this prognostic model exclusively utilizes TAM marker genes, excluding other potentially important tumor microenvironment characteristics (e.g., cancer cell intrinsic genes, other immune cell features, or clinical covariates). Additionally, TAM marker genes may lack purity, including redundant genes like *HBA2*. Although the model consistently outperformed multiple established benchmarks, absolute C-index indicates room for improvement in predictive capability. Future studies will focus on refining feature selection based on existing TAM characteristics. Integrating complementary multidimensional features with clinical variables to establish a multimodal integration framework holds promise for significantly enhancing prognostic accuracy and clinical utility.

## Figures and Tables

**Table 1:** Sample information.

Dataset Name	Sample	Gene	Censoring
TCGA	370	407	240 (64.9%)
OEP000321	158	510	102 (64.6%)
GSE14520	221	436	136 (61.5%)

**Table 2:** C-index comparison with other models.

Model Name	TCGA	GSE14520	OEP000321
ZZFormer	0.65437 ± 0.0589	0.64752 ± 0.0661	0.68922 ± 0.0729
DeepSurv	0.63301 ± 0.0505	0.60605 ± 0.0714	0.64029 ± 0.0794
DSM	0.60637 ± 0.0646	0.61035 ± 0.0750	0.64108 ± 0.0826
RSF	0.64876 ± 0.0606	0.61663 ± 0.0733	0.67183 ± 0.0713
GBM	0.65220 ± 0.0546	0.60228 ± 0.0603	0.65544 ± 0.0641

Highlighted in gray represents the model that achieves the highest C-index on each dataset

## Data Availability

The dataset provided in this study can be downloaded from the online website TCGA-LIHC: https://portal.gdc.cancer.gov/ (accessed on 1 July 2025), GEO: https://www.ncbi.nlm.nih.gov/geo/ (accessed on 1 July 2025) and NODE: https://www.biosino.org/node (accessed on 1 July 2025).

## Supplementary Materials

The following supporting information can be downloaded at: https://www.mdpi.com/article/10.3390/ijms26199825/s1.
